# Dynamic and Static Regulation of Nicotinamide Adenine Dinucleotide Phosphate: Strategies, Challenges, and Future Directions in Metabolic Engineering

**DOI:** 10.3390/molecules29153687

**Published:** 2024-08-03

**Authors:** Nana Ding, Zenan Yuan, Lei Sun, Lianghong Yin

**Affiliations:** 1 State Key Laboratory of Subtropical Silviculture, Zhejiang A&F University, Hangzhou 311300, China; 2023613031025yzn@stu.zafu.edu.cn (Z.Y.); 2022613032018@stu.zafu.edu.cn (L.S.); 2 Zhejiang Provincial Key Laboratory of Resources Protection and Innovation of Traditional Chinese Medicine, Zhejiang A&F University, Hangzhou 311300, China

**Keywords:** NADP(H), redox balance, static regulation, dynamic regulation, metabolic engineering

## Abstract

Reduced nicotinamide adenine dinucleotide phosphate (NADPH) is a crucial cofactor in metabolic networks. The efficient regeneration of NADPH is one of the limiting factors for productivity in biotransformation processes. To date, many metabolic engineering tools and static regulation strategies have been developed to regulate NADPH regeneration. However, traditional static regulation methods often lead to the NADPH/NADP^+^ imbalance, causing disruptions in cell growth and production. These methods also fail to provide real-time monitoring of intracellular NADP(H) or NADPH/NADP^+^ levels. In recent years, various biosensors have been developed for the detection, monitoring, and dynamic regulate of the intracellular NADP(H) levels or the NADPH/NADP^+^ balance. These NADPH-related biosensors are mainly used in the cofactor engineering of bacteria, yeast, and mammalian cells. This review analyzes and summarizes the NADPH metabolic regulation strategies from both static and dynamic perspectives, highlighting current challenges and potential solutions, and discusses future directions for the advanced regulation of the NADPH/NADP^+^ balance.

## 1. Introduction

Redox coenzyme NADPH is the reduced form of nicotinamide adenine dinucleotide phosphate (NADP^+^) [1]. It plays a crucial role in energy metabolism, providing high-energy electrons for antioxidant defense and reductive biosynthesis [2,3,4,5,6,7]. Metabolic pathway for NADP^+^ reduction include the oxidative pentose–phosphate pathway (oxPPP), the Entner–Doudoroff (ED) pathway, and the TCA cycle, with oxPPP being essential for maintaining a normal NADPH/NADP^+^ ratio [6]. However, in many cases, insufficient NADPH regeneration rate and availability often lead to cell death under high levels of reactive oxygen species (ROS) [8,9,10,11,12]. This also limits the production of high-value chemicals that require large amounts of this cofactor, such as amino acids [13,14,15,16], mevalonate [17], terpenes [18,19], and fatty-acid-based fuels [20]. Therefore, improving the flux and availability of NADPH has been a long-standing challenge in metabolic engineering [4]. Several strategies have been employed to overcome this limitation [21,22,23]. The most common static regulation strategy is to direct metabolic flux to endogenous NADPH biosynthesis pathways. For example, to improve the production of poly-3-hydroxybutyrate (PHB), Li et al. increased the supply of NADPH by overexpressing the endogenous genes *ppnK* and *zwf*, promoting the metabolic flux towards the PHB biosynthesis pathway [24]. Alternatively, NADPH can be increased through heterologous expression. For example, Lee et al. enhanced NADPH regeneration in *Escherichia coli* by expressing isocitrate dehydrogenases (IDHs) from *Corynebacterium glutamicum* and *Azotobacter vinelandii* [25]. Other strategies include reducing or knocking out competing pathways for NADPH regeneration [26], modifying cofactor preference through protein engineering [27], replacing NADPH-dependent enzymes with NADH-dependent ones [28], promoter/RBS engineering [29,30], and photo- or electro-chemical methods [31].

However, due to the varying intracellular NADPH levels required at different culture times, static regulation strategies often lead to an imbalance of NADPH/NADP^+^ as they cannot adjust intracellular NADPH levels in real time. To address this issue, leveraging the natural dynamic adjustment of intracellular NADPH levels due to the cyclicity of the ED pathway can be beneficial [32,33,34,35,36]. This is especially interesting in some *Pseudomonadaceae* and *Burkholderiaceae* in which the 6-phosphogluconate dehydrogenase encoded by the *gnd* gene is absent [37]. In the absence of the *gnd* gene, NADP^+^ is not reduced in the PPP. Thus, the ED pathway becomes the main source of NADPH, and the more cyclical the ED pathway is, the higher the amount of reduced NADP^+^ increases to the detriment of ATP production [37]. It was demonstrated that the cyclicity of the ED pathway is greater in the stationary growth phase in which the production of rhamnolipids and polyhydroxyalkanoates occurs than in the cell growth phase [37,38]. Besides, the expression of *gnd* genes from *E. coli* or *Pseudomonas putida*, which have different specificities for NAD^+^ or NADP^+^, in a PHA-producing *Pseudomonas* lacking *gnd* affected either cell growth or the production of polyhydroxyalkanoates, indicating that dynamic adjustment of the cyclicity of the ED pathway may represent a more effective strategy in some bacteria to transition between NADPH demands in the cell growth phase and the polyhydroxyalkanoate production phase [39]. These results suggest that cyclicity in the ED pathway may represent a natural mechanism of dynamic regulation of NADPH supply.

To effectively implement strategies for dynamically regulating NADPH levels, it is crucial to thoroughly understand the mechanisms of NADPH generation in bacteria. The current metabolic model of *P. putida* KT2440 assumes that glucose-6-phosphate dehydrogenase (G6PDH) exclusively uses NADP^+^ as a cofactor [40,41,42]. However, Olavarria et al. indicates that the G6PDH encoded by the *zwf-1* gene (PputG6PDH-1) actually produces approximately 1/3 mol NADPH and 2/3 mol NADH during the oxidation of glucose-6-phosphate, and 6-phosphogluconate dehydrogenase also generates NADH [43]. This suggests that G6PDH in *P. putida* KT2440 can recognize both NADP^+^ and NAD^+^ [43]. Therefore, it is crucial to revise the stoichiometric matrix of the metabolic model and consider the physiological concentrations of metabolites to accurately estimate the actual ratio of NADH to NADPH [43]. These insights contribute to optimizing metabolic engineering strategies based on *P. putida* KT2440, thus enhancing the understanding of bacterial metabolic adaptation under varying environmental conditions [43]. In this context, understanding how bacteria balance the consumption and generation of NADH and NADPH is particularly important. The three G6PDH isoenzymes in *P. putida* KT2440, encoded by the *zwfA*, *zwfB*, and *zwfC* genes, exhibit different specificities for NAD^+^ and NADP^+^ [44]. Research has shown that these isoenzymes play a crucial role in maintaining redox balance during the metabolism of various carbon sources, especially in the metabolism of fructose and ribose, where almost all carbon flux passes through the G6PDH reaction [44]. Bacteria that rely on the EMP pathway enhance NADPH supply by directing flux through the PP or ED pathways via G6PDH, while those that depend on the ED pathway achieve this balance through isoenzymes with distinct cofactor specificities [44]. This indicates that the different specificities of G6PDH isoenzymes are closely related to bacterial metabolic strategies under various environmental conditions, reflecting an evolutionary adaptation to balance the production of NADPH and NADH, thus providing a theoretical basis for the dynamic regulation of the NADPH/NADP^+^ balance.

Additionally, a genetically encoded biosensor can be used to construct a dynamic NADPH regulation system, allowing real-time monitoring of intracellular NADP(H) redox status and subsequently regulating the NADPH/NADP balance, which is a determinant of cellular energy y availability [45,46]. Genetically encoded biosensors can measure and manipulate NADPH metabolism [45]. Studies have shown that the transcription factor SoxR biosensor specifically responds to NADPH/NADP^+^, making it a useful tool for investigating NADPH-related processes in *E. coli* and laying a solid foundation for dynamically regulating NADPH production or consumption [3]. However, the SoxR biosensor can only regulate the NADP(H) redox balance in *E. coli*. To evaluate the NADP(H) redox status in all organisms, Pamela et al. developed a ratiometric biosensor named NERNST, which real-time monitors the NADP(H) redox status based on a redox-sensitive green fluorescent protein (roGFP2) and an NADPH thioredoxin reductase C module [7]. NERNST can assess the NADPH/NADP balance in organisms and holds various potential applications in metabolic engineering, biotechnology, and synthetic biology research [7]. Therefore, genetically encoded biosensors have great potential in regulating the dynamic balance of NADPH/NADP^+^ within cells.

This review aims to discuss and summarize the static and dynamic regulation strategies of NADPH levels and the NADPH/NADP^+^ ratio. We also discuss the impact of these regulation strategies on cellular metabolism, identify existing problems and challenges, and finally, provide an outlook on future directions for the advanced regulation of NADPH.

## 2. Static Regulation Strategies for NADPH Regeneration

**Figure 1:**
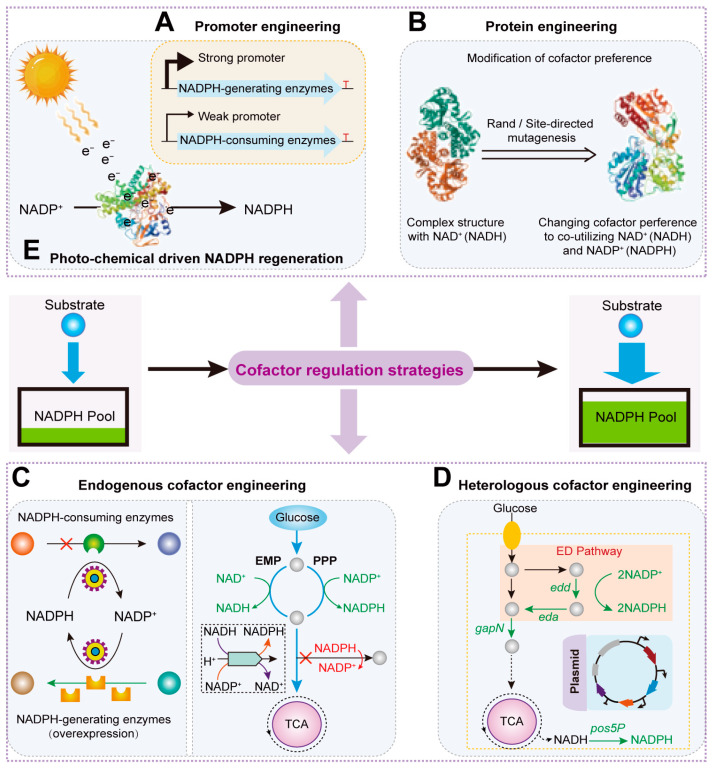
The static regulation strategies for increasing the NADPH pool. (**A**) The promoter engineering to control the NADPH level. (**B**) The protein engineering to modify the cofactor preference. The endogenous (**C**) and heterologous (**D**) cofactor engineering to improve the NADPH level. (**E**) The photo-chemical method to drive the NADPH regeneration.

Although NADPH regeneration can be achieved through external regulation methods, such as adding electron donors or acceptors or adjusting dissolved oxygen concentration, the primary method currently relies on endogenous regulation by genetically engineering NADPH metabolic pathways within the organism. In microorganisms, NADPH is mainly produced by central carbon metabolic pathways, with the pentose phosphate pathway (PPP) being the primary source. In the PPP, NADP^+^ is reduced to NADPH by the enzymes Zwf and Gnd, providing reducing power for intracellular enzymatic reactions. In the Entner–Doudoroff pathway, the NADP+ is reduced in the same reaction of the PPP, which is catalyzed by glucose-6-phosphate dehydrogenase (Zwf). Additionally, in the EMP pathway, NADPH is only produced under specific conditions where glyceraldehyde-3-phosphate dehydrogenase depends on NADP^+^. Furthermore, the isocitrate dehydrogenase reaction in the TCA cycle are also significant sources of NADPH. Current static regulation strategies for NADPH regeneration focus mainly on the following aspects (Figure 1): (1) Promoter and RBS engineering to precisely regulate the expression of NADP(H)-dependent enzymes; (2) Protein engineering to modify the cofactor preference of dependent enzymes; (3) Endogenous cofactor engineering to control the expression of genes involved in NADPH consumption and regeneration; (4) Heterologous cofactor engineering to supplement the NADPH regeneration system; (5) (Photo- or electro-) chemical methods driven by solar energy, electron transfer, or chemical catalysts for NADPH regeneration.

### 2.1. Promoter and RBS Engineering Strategies to Enhance NADPH Regeneration

Currently, microbial bioprocesses are commonly used to produce various bulk chemicals. Since the biosynthesis of target chemicals largely requires NADPH, the titer and yield of these chemicals are often limited by insufficient NADPH supply. This limitation is primarily due to the inefficient expression of NAD(P)H-dependent enzymes in the biosynthetic pathways. To address this problem, researchers have employed promoter engineering to direct more carbon flux toward the PPP pathway, thereby enhancing NADPH regeneration and facilitating the efficient biosynthesis of chemicals (Figure 1A). For example, Kobayashi et al. replaced the promoter of the glucose 6-phosphate isomerase gene *pgi* with the anaerobic-specific promoter of the lactate dehydrogenase gene (*ldhA*) [47]. This increased the proportion of carbon flux entering the PPP from 39% to 83% under aerobic conditions, thereby enhancing NADPH synthesis [47]. As a result, the titer, yield, and productivity of 1,5-diaminopentane increased by 4.6-, 4.4-, and 2.6-fold, respectively [47]. Qin et al. first reduced the expression of the *pgi* gene by replacing its natural promoter with a weaker one [29]. Then, they increased the expression of glucose-6-phosphate dehydrogenase (Zwf) and 6-phosphogluconate dehydrogenase (Gnd) by replacing their natural promoters with stronger ones and overexpressing cytosolic aldehyde dehydrogenase (ALD) [29]. This ultimately increased NADPH supply, raising the titer of 3-hydroxypropionic acid (3-HP) to 864.5 mg/L [29]. Li et al. selected five promoters of varying strengths from the Anderson promoter library to replace the promoter of the *zwf* gene in the genome, thereby enhancing PPP flux and increasing NADPH levels [48]. Among these strains, BP10BF accumulated 11.2 g/L of mevalonate (MVA) after 72 h of fermentation, with a molar conversion rate of 62.2% from glucose [48].

In addition, RBS engineering has also demonstrated significant potential in fine-tuning gene expression. For example, Xiong et al. combined alcohol dehydrogenase (ADH) and cyclohexanone monooxygenase (CHMO) to develop an adaptive NADPH regeneration system [49]. By designing RBS sequences to achieve optimal NADPH levels, they obtained a variant that produced ε-caprolactone with a yield of 0.80 mol/mol using 60 mM cyclohexanol as the substrate [49]. Therefore, promoter and RBS engineering are advantageous strategies for enhancing NADPH regeneration and hold significance for the efficient biosynthesis of chemicals.

### 2.2. Protein Engineering Strategies to Enhance NADPH Levels

With the advancement of structural biotechnology, an increasing number of three-dimensional enzyme structures have been elucidated. Combining this with gene-mutation techniques allows for modifications of enzyme biocatalysis related to cofactors [50]. Research progress has shown that using site-directed mutagenesis to alter key enzyme sites, thereby changing their cofactor affinity or preference, is an important method for enhancing the production of target metabolites (Figure 1B) [51,52]. For example, Zhang et al. used an Ser361Phe mutation in the *gnd* gene to reduce the allosteric regulation of the enzyme, directing more carbon flux into the PPP pathway [53]. They also introduced an Ala243Thr mutation in the *zwf* gene, which increased the affinity of glucose-6-phosphate dehydrogenase (G6PDH) for NADP^+^ [53]. This resulted in the construction of an L-proline-producing mutant strain with enhanced NADPH supply, increasing its yield to 6.17 ± 0.59 g/L [53]. Similarly, Liu et al. performed site-directed mutagenesis on the *zwf* and *gnd* genes in the PPP pathway and heterologously expressed *gapC* and *pntAB* to create the engineered strain YM6, which increased NADPH supply by 348.2% [54]. Consequently, the L-methionine titer increased by 64.1%, reaching 0.64 g/L [54]. Jia et al. used site-directed mutagenesis to enhance the alcohol dehydrogenase (ADH) activity of the reductive aminase from *Aspergillus oryzae* (*Asp*RedAm), which shows a preference for NADPH in reductive amination reactions [55]. Molecular docking revealed that the N93A mutation (asparagine to alanine) enhanced thermal stability [55]. The smaller alanine side chain in the substrate binding pocket reduced steric hindrance, doubling the catalytic efficiency and thereby strengthening NADPH synthesis [55]. Additionally, there have been reports of obtaining NADPH-dependent variants through non-standard saturation mutagenesis and 96-well plate screening methods [27]. For example, Huang et al. developed the NAD(P)-eliminated solid-phase assay (NESPA), which involved colorimetric screening of colonies grown on agar plates [27]. After six rounds of directed evolution, they obtained an optimal 6-phosphogluconate dehydrogenase mutant with a 50-fold increase in catalytic efficiency, enhancing NADPH regeneration [27].

Compared to site-directed mutagenesis, large random library mutagenesis has limited applications in specifically altering enzyme cofactor preferences due to the extensive screening required [50]. However, high-throughput screening using computational methods has made it possible to enhance enzyme specificity and catalytic efficiency [56,57,58,59]. For example, Calzadiaz-Ramirez et al. leveraged natural selection in NADPH-deficient auxotrophic strains to design and optimize a formate dehydrogenase (FDH) variant library for NADP^+^ specificity and kinetic efficiency [60]. They identified variants that could support efficient NADPH regeneration in vivo [60]. Through molecular dynamics (MD) simulations and steady-state kinetic analyses, they identified multiple residues likely affecting enzyme activity and NADP^+^ specificity [60]. Systematic mutagenesis of these residues generated a library of over 10^6^ variants, which was introduced into an NADPH-auxotrophic *E. coli* strain [60]. They ultimately isolated several enzyme variants that supported efficient NADPH regeneration, with catalytic efficiency and specificity improved by 5- and 14-fold, respectively, compared to the previously best-designed enzyme [60]. Additionally, due to the low sequence homology (<26%) of NADPH-dependent carboxylic acid reductases (CARs) with other subgroups, most CAR engineering efforts still rely on random mutagenesis [61]. Therefore, Schwendenwein et al. developed an amino benzamidoxime (ABAO)-mediated high-throughput assay (HTA) driven by random mutagenesis for screening mutant libraries in whole-cell systems [61]. They successfully obtained a CAR variant (Q283P) with improved catalytic efficiency and a 9-fold increased affinity for 2-methoxybenzoic acid [61]. This was the first time a CAR library comprising thousands of clones was screened using a robot-assisted platform [61]. These achievements make cofactor preference engineering a routine task rather than a daunting effort, continuing to optimize current strategies for regulating NADPH systems.

### 2.3. Endogenous Cofactor Engineering Strategies to Regulate NADPH Consumption and Regeneration

Sufficient NADPH supply is essential for achieving high titers of chemicals. The regulation methods of endogenous cofactor systems primarily include knocking out competitive NADPH-consuming pathways and enhancing endogenous NADPH-generating pathways (Figure 1C). Knocking out or inhibiting competitive NADPH-consuming pathways has been applied to engineering *R. capsulatus* for bisabolene production, engineered *Saccharomyces cerevisiae* for β-carotene production, and engineered *E. coli* for 4-hydroxyphenylacetic acid (4HPAA) production. For bisabolene production, CRISPR/Cas12a technology was used to knock out the NADPH-dependent genes *phbC* and *gltBD*, increasing NADPH availability and achieving a bisabolene titer of 390.3 mg/L [62]. To increase NADPH concentration for β-carotene production, CRISPR-Cas9 technology was employed to knock out the NADPH-dependent aldehyde reductase gene *yjgB* and overexpress the native genes *mdh*, *pntAB*, *sthA*, and *nadK* (*yfjB*) from *S. cerevisiae*, along with the heterologous gene *tPOS5p*. This led to a β-carotene titer of 2579.1 mg/L in a 5 L bioreactor [63]. For efficient 4HPAA biosynthesis, Shen et al. used the clustered regularly interspaced short palindromic repeats interference (CRISPRi) screening (CECRiS) method to inhibit all NADPH-consuming enzyme-encoding genes in *E. coli*. Through CRISPRi screening, six NADPH-consuming enzyme-encoding genes were identified, and their inhibition increased NADPH availability, raising the 4HPAA titer from 6.32 g/L to 7.76 g/L [26]. Additionally, knocking out the gene *pgi* to disrupt the competitive EMP pathway and redirect more carbon flux into the PPP pathway is another important strategy for enhancing NADPH supply [64,65].

NADPH levels can also be increased by enhancing endogenous pathways that produce NADPH [66]. Numerous studies have shown that overexpressing the transhydrogenase gene *pntAB* enhances NADPH supply, resulting in increased titers of various compounds: Ferulic acid (FA) from 130 mg/L to 212 mg/L [67], 2-Ketoisovalerate biosynthesis by 11% [68], and L-arginine production to 38.9 g/L [69]. Other studies have demonstrated that overexpressing *zwf* and/or *glk* and *gnd* genes increased the NADPH/NADP^+^ ratio by 2.3-fold [70], NADPH levels by 4.5-fold [71] and 20.55% [72], L-cysteine titer to 2.11 g/L [73], and achieved efficient biosynthesis of α-Farnesene in *Pichia pastoris* [74]. Furthermore, to enhance NADPH supply, Yin et al. overexpressed the NAD^+^ kinase gene *ppnK* in L-Ile-producing *C. glutamicum* IWJ001, increasing the NADPH/NADP^+^ ratio in the L-Ile biosynthetic pathway. Fed-batch bioprocess achieved L-Ile titer and yield of 32.3 g/L and 0.116 g/g glucose, respectively [75]. Moreover, NADP^+^-dependent isocitrate dehydrogenase (IDH) is a key factor in regulating lipid biosynthesis. Li et al. overexpressed IDH, enhancing NADPH supply and achieving a lipid titer of 234.56 mg/L [76]. These examples illustrate that targeted regulation of endogenous cofactor systems by modulating complementary cofactor-producing pathways is a straightforward method to promote NADPH regeneration for the efficient production of chemicals and biofuels.

### 2.4. Heterologous Cofactor Engineering Strategies to Supplement NADPH Regeneration Systems

Recent advances in metabolic engineering and synthetic biology have made it possible to regulate NADPH regeneration by introducing heterologous NADPH regeneration systems [77]. For example, Hoffmann et al. improved NADPH supply by co-expressing the NADP^+^-dependent glyceraldehyde 3-phosphate dehydrogenase (GapN) from *Streptococcus* mutans and the native NAD^+^-dependent GapA enzyme, which coupled the improved flux of the EMP pathway with NADPH formation, ultimately increasing the lysine titer to 14 mM [78]. To optimize the heterologous expression of maltose α-amylase (AmyM) from *Geobacillus thermoglucosidasius* in *Bacillus subtilis* WB800, Chen et al. introduced the hemoglobin gene *vgb* from *Vitreoscilla* into the AmyM recombinant strain, enhancing intracellular NADPH and NADP^+^ levels and increasing its expression by 204.08% [79]. To adjust the competitive balance between glucose and xylose co-metabolism in lignocellulosic hydrolysates and increase ethanol titer, Qiu et al. knocked out ZWF1 and replaced the endogenous NAD^+^-dependent glyceraldehyde-3-phosphate dehydrogenase (GAPDH) gene *TDH3* with the heterologous NADP^+^-GAPDH genes *GDH*, *gapB*, and *GDP1*. This reconstructed the NADPH regeneration pathway, resulting in a 1.6-fold increase in xylose utilization after glucose depletion [80]. Hao et al. introduced the ED pathway from *Zymomonas mobilis* into *E. coli* (Figure 1D). Under the control of a strong P_trc_ promoter, they heterologously expressed the genes *edd* and *eda*, encoding 6-phosphogluconate dehydratase and 2-keto-3-deoxygluconate-6-phosphate aldolase, respectively. NADPH levels increased by 45.24% and L-valine production increased by 22.13%, reaching 21.25 g/L [81].

### 2.5. Photo- or Electrochemical Methods Driven NADPH Regeneration

With the increasing number of chemical catalysts being reported, NADPH regeneration in biosynthetic systems can be achieved through chemical methods such as inorganic salts and transition metal complexes [31]. Recently, a novel artificial metalloenzyme designed for NADPH regeneration utilizing ADH has been developed [82]. In metal oxides (Fe_2_O_3_ or MgO), electrons efficiently reduce NADP^+^ to NADPH [83]. However, problems such as lack of product purity and incompatibility between NADPH and chemical catalysts have limited the application of these methods [31]. To solve the problem, Kadowaki et al. developed a nanostructured Ni-Cu_2_O-Cu heterolayer material that promotes the electrochemical regeneration of NADPH [84]. By sputtering nickel onto a copper oxide electrode, a unique surface morphology was created, leading to high product selectivity and allowing for the extensive conversion of NADP^+^ to NADPH [84]. Constructing highly selective electrochemical methods further enhances NADPH regeneration. Additionally, photochemistry offers a solar-powered method for NADPH regeneration [85]. Ma et al. engineered photosynthetic bacteria such as *Rhodobacter sphaeroides* by heterologously expressing ADH, establishing a photo-driven NADPH regeneration system for synthesizing chiral alcohols (Figure 1E) [85]. The system successfully reduced the model substrate 3′-chlorophenylacetone to (R)-1-(3-chlorophenyl) ethanol with an enantiomeric excess greater than 99% [85]. Compared to wild-type photosynthetic bacteria, the whole-cell catalyst based on engineered bacteria bypassed the limitations of enzyme selectivity, facilitating NADPH regeneration.

## 3. Strategies for Dynamic Regulation of NADPH/NADP^+^ Ratio Based on Regulatory Element Libraries and Genetically Encoded Biosensors

**Figure 2:**
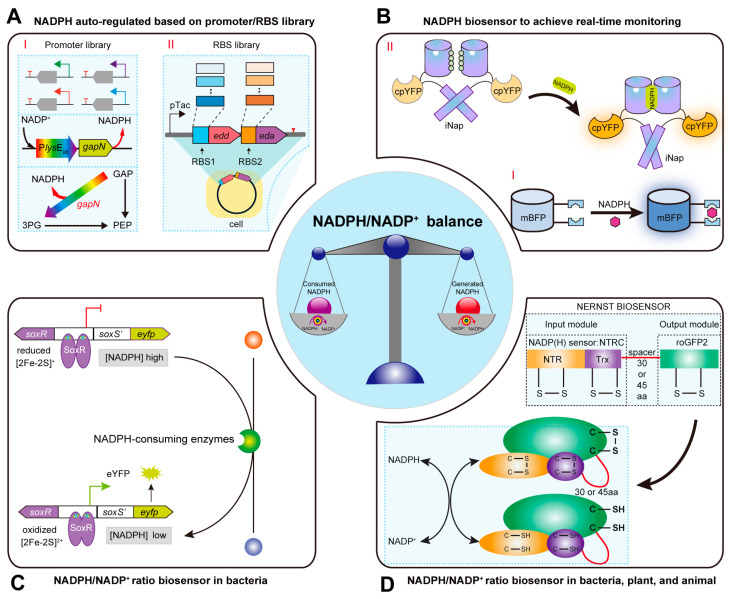
The dynamic regulation strategies for regulating the NADPH/NADP^+^ balance. (**A**) The auto-regulation of NADPH level based on the design of promoter/RBS library. (**I**) Schematic diagram of the intracellular NADPH dynamic regulation strategy based on promoter libraries. GAP, glyceraldehyde 3-P; 3PG, 3-P Glycerate; PEP, phosphoenolpyruvate; *gapN*, gene encoding glyceraldehyde-3-phosphate dehydrogenase N; P*lys*E_lib_, lysE promoter library; (**II**) Schematic diagram of the intracellular NADPH dynamic regulation strategy based on RBS libraries. *edd*, gene encoding 6-phosphogluconate dehydratase; *eda*, gene encoding 2-keto-3-deoxygluconate-6-phosphate (KDPG) aldolase. (**B**) The construction of mBFP and iNap biosensors to realize the detection and monitoring of NADPH levels in real time. (**I**) Working mechanisms of mBFP for detecting NADPH; (**II**) The working mechanism of iNap sensors. CpYFP, circularly permuted YFP. The construction of NADPH/NADP^+^ ratio biosensor to control the redox balance in bacteria (**C**) and plant and animal cells (**D**).

In typical metabolic pathways, the NADPH/NADP^+^ ratio reflects changes in the cellular redox state, which are associated with cell growth, physiology, and chemical production [86]. The intracellular NADPH/NADP^+^ ratio ranges from 1.05 to 58.8 [9], indicating a reductive state. However, metabolic flux can lead to an imbalance in the NADPH/NADP^+^ ratio, resulting in metabolic energy depletion, cell damage, and even metabolic shock [2,9,50,87]. Fortunately, an imbalanced redox state can be restored by dynamically regulating the NADPH/NADP^+^ ratio through several strategies (Figure 2): (1) Designing a library of regulatory elements to dynamically regulate the expression of NADPH-dependent genes; (2) Constructing NADPH biosensors to monitor intracellular NADPH changes in real time; (3) Developing genetically encoded biosensors that respond to NADPH/NADP^+^, providing valuable tools to monitor dynamic changes in NADPH in living cells and gain new insights into cellular metabolism [88].

### 3.1. Constructing Promoter and RBS Libraries for Dynamic Regulation of the NADPH Pool

Static regulation of the NADPH pool struggles to meet the real-time needs of cell growth and chemical synthesis, making it unsuitable for maintaining redox homeostasis in the intracellular environment. Controllable NADPH regeneration rates are key to maximizing the titers of chemical production [89]. In the 2-Pyrone-4,6-dicarboxylic acid (PDC) metabolic pathway, one molecule of NADP^+^ is converted to NADPH, which can disrupt the NADPH/NADP^+^ balance in the cell [90]. To dynamically regulate the NADPH pool, Luo et al. replaced the native promoter with the strong Trc promoter to overexpress soluble pyridine nucleotide transhydrogenase gene *sthA* in *E. coli*, enhancing its catalytic activity. SthA is responsible for re-oxidizing excess NADPH to NADP^+^ [90]. Then, three silent point mutations (C15T, T18C, C21T) were introduced into the chromosomal *sthA* gene to dynamically regulate the NADPH pool by interfering with small regulatory RNA-based *sthA* expression, providing insights into the impact of redox cofactor balance on PDC production [90]. Liu et al. constructed a lysine concentration-responsive promoter library to regulate the expression of non-phosphorylating NADP^+^-dependent glyceraldehyde-3-phosphate dehydrogenase, dynamically optimizing the intracellular NADPH pool, resulting in a lysine titer of 223.4 ± 6.5 g/L in the engineered strain (Figure 2A(I)) [91]. Due to endogenous transcriptional, translational, and allosteric regulation layers, engineering the natural *E. coli* ED pathway cannot dynamically control its NADPH regeneration rate [89]. Ng et al. used an RBS library calculator to efficiently explore the five-dimensional enzyme expression space and conducted 40 rounds of MAGE to design and construct improved pathway variants (Figure 2A(II)) [89]. They then screened 624 pathway variants using NADPH-dependent blue fluorescent protein (mBFP) and further characterized 22 variants to determine the relationship between enzyme expression levels and NADPH regeneration rate [89]. The optimized ED pathway ultimately increased NADPH-dependent mBFP fluorescence by 25-fold and improved carotenoid production titers by 97% [89]. Therefore, combining systematic metabolic engineering with dynamic regulation can be widely applied to construct efficient microbial cell factories for producing valuable products.

### 3.2. Constructing Biosensors for Real-Time Monitoring of Intracellular NADP(H) Levels

Recently, researchers have discovered that the metagenome-derived mBFP, a member of the short-chain dehydrogenase/reductase family, does not exhibit intrinsic fluorescence when heterologously expressed in *C. glutamicum*. However, it produces fluorescence proportional to the NADPH concentration upon specific binding to NADPH (Figure 2B(I)) [92,93]. Compared to kit-based methods, mBFP detection offers advantages such as speed, sensitivity, and low cost, making it an effective tool for measuring intracellular NADPH levels [93,94,95]. For example, Goldbeck et al. treated cells with cetyltrimethylammonium bromide to increase cell membrane permeability, allowing extracellular NADPH at gradient concentrations to diffuse into the cells. By measuring the resulting mBFP fluorescence intensity, they established a standard curve for detecting intracellular NADPH concentrations [92]. Thus, the mBFP biosensor holds promise for real-time quantitative detection of NADPH levels within cells.

In the metabolic engineering of microbial cell factories, traditional static regulation strategies often disrupt the intracellular NADPH/NADP^+^ balance, hindering the coordination of cell growth and product synthesis. Therefore, researchers have recently designed and constructed high-performance NADPH and NADP^+^ biosensors that can monitor the dynamic changes of intracellular NADPH and NADP^+^ in real time, enabling the regulation of metabolic pathways [86,96]. For example, Tao et al. developed the iNap biosensor by mutating the binding pocket amino acids of the NADH biosensor SoNar based on the structural similarity between NADPH and NADH, to achieve a specific response to NADPH (Figure 2B(II)) [88]. This biosensor is now widely used for the dynamic regulation of NADPH levels in eukaryotic cells. For example, Lim et al. introduced the iNap biosensor into *Arabidopsis* to monitor dynamic changes in NADPH levels [97]. Under illumination, photosynthesis is related to the redox state of the NAD(P)H pool, which is connected by several subcellular compartments through the malate-OAA shuttle [97]. They found that when glycine decarboxylation was inhibited, photosynthesis increased matrix NADPH. This study provided a valuable tool for real-time monitoring of dynamic changes in reducing equivalents without damaging plant tissues [97]. Additionally, Moon et al. combined dynamic experimental data obtained from the iNap biosensor with an analysis of central carbon metabolism under perturbation to establish a mathematical model quantifying mitochondrial NADPH concentrations at different hydrogen peroxide production rates [98]. Their results showed that excessive mitochondrial hydrogen peroxide production reduces the mitochondrial NADPH pool, subsequently activating the PPP pathway and glucose degradation to maintain the NADPH pool [98]. The quantitative approach offers insights into mitochondrial NADPH metabolism during oxidative stress.

To monitor intracellular NADP^+^ levels in real time, Zhao et al. developed an NADP^+^ biosensor by incorporating the reporter protein ketopantoate reductase (KPR) between a Förster resonance energy transfer (FRET) pair, cyan fluorescent protein (CFP), and yellow fluorescent protein (YFP). This biosensor achieves a specific response to NADP^+^. However, the dynamic range and sensitivity of the biosensor still need improvement [99]. To enhance the biosensor’s sensitivity to NADP^+^, Cameron et al. developed an Apollo sensor for NADP^+^ (Apollo NADP^+^) for multiparameter imaging based on the NADP^+^-induced homodimerization of G6PD, which triggers fluorescence resonance energy transfer [100]. These biosensors consist of fluorescent proteins and inactive G6PD and exhibit high sensitivity to NADP^+^ [100]. Nonetheless, the narrow dynamic range (15–20%) caused by slight conformational changes in Apollo NADP^+^ still limits its broad application [100]. Moreover, these biosensors primarily focus on monitoring and imaging NADPH and NADP^+^ levels in cells, making it challenging to dynamically monitor changes in the intracellular NADPH/NADP^+^ ratio.

### 3.3. Constructing NADPH/NADP^+^ Ratio Biosensors for Dynamic Regulation of Redox State

To monitor intracellular NADPH/NADP^+^ levels, studies have identified the transcription factor SoxR in *E. coli*, which is a homodimer containing two iron-sulfur clusters ([2Fe-2S]) [101]. SoxR specifically responds to the NADPH/NADP^+^ ratio through structural changes between the oxidized and reduced forms of the [2Fe-2S] cluster, thereby activating the expression of the *soxS* gene controlled by P_soxS_ with high sensitivity (Figure 2C) [102]. Based on this discovery, Siedler et al. developed the pSenSox sensor to respond to NADPH/NADP^+^ in *E. coli*, enabling high-throughput screening of NADPH-dependent ADH with optimal activity [102]. Subsequently, Spielmann et al. used the pSenSox sensor and fluorescence-activated cell sorting (FACS) to screen an ADH library from *Lactobacillus brevis* (LbADH) in *E. coli*, identifying LbADH variants with improved catalytic performance [103]. Additionally, Zhang et al. utilized the natural yeast Yap1p regulatory pathway by fusing the TRX2 promoter with green fluorescent protein, developing an NADPH/NADP^+^ biosensor in yeast [104]. The biosensor combined with the expression of dosage-sensitive genes, successfully screened cells with high NADPH/NADP^+^ ratios [104]. To explore the diversity of compartmental redox states and monitor yeast production processes in real time, researchers designed compartment-targeted redox biosensors in *S. cerevisiae*, providing a standard for dynamically monitoring the redox states of organelles under different stress conditions [105]. However, the biosensor is functional only in yeast cells, limiting its application. To overcome this limitation, Molinari et al. fused NADPH-dependent thioredoxin reductase C (NTRC) with redox-sensitive green fluorescent protein (roGFP2) to construct the NERNST biosensor, which responds to intracellular NADPH/NADP^+^ ratios in bacteria, plants, and animal cells (Figure 2D) [7]. The NERNST biosensor allows dynamic monitoring of redox states through real-time imaging or fluorescence spectroscopy [7].

## 4. Conclusions and Perspective

Although genetically encoded biosensors can dynamically regulate the intracellular NADPH redox state [96,106], these biosensors typically only control either the generation or consumption of NADPH, making it difficult to autonomously regulate the activation and inhibition of NAD(P)H- or NAD(P)^+^-dependent oxidoreductases. This bifunctional regulation—dynamically controlling both NADPH-generating and NADPH-consuming enzymes—is crucial for maintaining normal cellular metabolism. Currently, there is a lack of precise and dynamic bifunctional NADPH regulation methods in cells. Thus, designing NADPH bifunctional biosensors is key to solving this challenge. Recently, some regulatory elements like CRISPRi have been reported to construct bifunctional regulation systems [107,108]. However, the expression of CRISPRi not only consumes cellular resources but also reduces the responsiveness and robustness of the cell’s global regulatory system. Therefore, developing a resource-efficient and effective molecular switch is essential for designing bifunctional regulation systems. Studies have shown that antisense transcription can affect gene expression by interfering with sense-strand transcription through antisense RNA (asRNA), making it an ideal tool for designing bifunctional regulation systems [109]. For example, Xu et al. developed a pyruvate-responsive bifunctional biosensor based on antisense transcription and the PdhR sensor from *E. coli*, dynamically regulating gluconate production in *B. subtilis* and increasing gluconate titer from 207 mg/L to 527 mg/L [110]. Therefore, utilizing antisense transcription and cofactor biosensors is an ideal approach for constructing NADPH bifunctional biosensors, which are crucial for the precise and dynamic regulation of the NADPH/NADP^+^ balance.

To regulate the NADPH/NADP^+^ balance in cells, many studies have employed metabolic engineering and synthetic biology strategies to control the intracellular NADPH/NADP^+^ ratio [111,112]. New techniques for regulating the NADPH/NADP^+^ balance have been developed, including promoter engineering [90,91], systems metabolic engineering [113,114], and cofactor engineering [9,50]. However, due to the complexity of NADPH metabolism and function, existing strategies for regulating the NADPH/NADP^+^ balance are not effective at controlling the overall metabolic balance of intracellular cofactors. More importantly, the mechanisms underlying the dynamic regulation of NADPH/NADP^+^ balance are still unknown. Therefore, constructing NADPH-auxotrophic strains as sensor strains in cell factories and engineering NADP^+^-dependent enzymes can optimize cofactor redox balance [1,56,115]. Additionally, by gathering large datasets that link the characteristics of biological elements in regulatory strategies—such as promoter engineering, protein engineering, and systems metabolic engineering—with the NADPH/NADP^+^ ratio, artificial intelligence (AI) can potentially unravel the mechanisms of intracellular NADPH/NADP^+^ balance regulation [116]. In recent years, data-driven deep neural network models have made significant progress in multiple fields [117,118,119], such as precision prediction of protein structures and gene regulatory functions [57,120,121,122]. However, these models are often opaque black boxes, making it difficult to understand their internal mechanisms, which severely limits the analysis of gene regulatory patterns. To address the problem, Wei et al. developed the neural network interpretation algorithm NeuronMotif, which automatically infers and extracts gene regulatory sequence coding rules from neurons [123]. The algorithm can utilize neural networks to obtain understandable knowledge from massive data, helping to deeply understand the gene regulatory patterns of complex biological processes. This is significant for elucidating the mechanisms of intracellular NADPH/NADP^+^ balance and advancing knowledge in related interdisciplinary fields.

In conclusion, maintaining the NADPH/NADP^+^ balance is essential for cellular metabolism, cell growth, physiological functions, and chemical biosynthesis. Traditional static regulation methods are insufficient for dynamic control. Recent advances, including promoter engineering, protein engineering, and systems metabolic engineering, have improved regulation strategies. Genetically encoded biosensors enable real-time monitoring and dynamic regulation, but there is still a need for bifunctional biosensors that control both NADPH generation and consumption. Leveraging AI and large datasets to analyze regulatory mechanisms can provide deeper insights into maintaining the balance, optimizing microbial cell factories, and enhancing our understanding of cellular redox regulation.
